# Pandemic intake questionnaire to improve quality, effectiveness, and efficiency of outpatient neurologic and developmental care at the Kennedy Krieger institute during the COVID-19 pandemic

**DOI:** 10.3389/fresc.2022.934558

**Published:** 2022-10-06

**Authors:** Pooja Vedmurthy, Connor Murray, Belinda Chen, Akua Asiedu, Kristin Baranano, Mihee Bay, Harolyn Belcher, Vera Burton, Charles Conlon, Amena Fine, Ryan Gill, Jacqueline Harris, Khaylynn Hart, Shannon Inches, Jennifer Johnson, Eboni Lance, Paul H. Lipkin, Deepa U. Menon, Tiffany McIntyre, Meghna Rajaprakash, Albert Recio, Harvey S. Singer, Lindsay Smegal, Constance L. Smith-Hicks, Hilary Vernon, Anna Maria Wilms Floet, Joyce Wong, Karina Yelin, Mary L. O’Connor Leppert, T. Andrew Zabel, Anne M. Comi

**Affiliations:** ^1^ Department of Neurology, Kennedy Krieger Institute, Baltimore, MD, United States; ^2^ Department of Neurology and Developmental Medicine, Kennedy Krieger Institute, Baltimore, MD, United States; ^3^ Center for Development and Learning, Kennedy Krieger Institute, Baltimore, MD, United States; ^4^ Department of Neurology, Johns Hopkins University School of Medicine, Baltimore, MD, United States; ^5^ Department of Pediatrics, Johns Hopkins University School of Medicine, Baltimore, MD, United States; ^6^ Center for Diversity in Public Health Leadership Training, Kennedy Krieger Institute, Baltimore, MD, United States; ^7^ Office for Health Equity, Inclusion and Diversity, Kennedy Krieger Institute, Baltimore, MD, United States; ^8^ Department of Neuropsychology, Kennedy Krieger Institute, Baltimore, MD, United States; ^9^ Center for Autism and Related Disorders, Kennedy Krieger Institute, Baltimore, MD, United States; ^10^ International Center for Spinal Cord Injury, Kennedy Krieger Institute, Baltimore, MD, United States; ^11^ Department of Physical Medicine and Rehabilitation, Johns Hopkins University School of Medicine, Baltimore, MD, United States; ^12^ Department of Genetic Medicine, Johns Hopkins University School of Medicine, Baltimore, MD, United States; ^13^ Department of Psychiatry and Behavioral Sciences, Johns Hopkins University School of Medicine, Baltimore, MD, United States

**Keywords:** pandemic (COVID-19), pediatrics - children, rehabilitation, SARS – CoV – 2, neurodevelomental disorders

## Abstract

**Background:**

The COVID-19 pandemic uniquely affects patients with neurologic and developmental disabilities at the Kennedy Krieger Institute. These patients are at increased risk of co-morbidities, increasing their risk of contracting COVID-19. Disruptions in their home and school routines, and restrictions accessing crucial healthcare services has had a significant impact.

**Methods:**

A Pandemic Intake questionnaire regarding COVID-19 related medical concerns of guardians of patients was distributed using Qualtrics. Data from May-December 2020 were merged with demographic information of patients from 10 clinics (Center for Autism and Related Disorders (CARD), Neurology, Epigenetics, Neurogenetics, Center for Development and Learning (CDL) Sickle Cell, Spinal Cord, Sturge-Weber syndrome (SWS), Tourette's, and Metabolism). A provider feedback survey was distributed to program directors to assess the effectiveness of this intervention.

**Results:**

Analysis included responses from 1643 guardians of pediatric patients (mean age 9.5 years, range 0–21.6 years). Guardians of patients in more medically complicated clinics reported perceived increased risk of COVID-19 (*p* < 0.001) and inability to obtain therapies (*p* < 0.001) and surgeries (*p* < 0.001). Guardian responses from CARD had increased reports of worsening behavior (*p* = 0.01). Providers increased availability of in-person and virtual therapies and visits and made referrals for additional care to address this. In a survey of medical providers, five out of six program directors who received the responses to this survey found this questionnaire helpful in caring for their patients.

**Conclusion:**

This quality improvement project successfully implemented a pre-visit questionnaire to quickly assess areas of impact of COVID-19 on patients with neurodevelopmental disorders. During the pandemic, results identified several major areas of impact, including patient populations at increased risk for behavioral changes, sleep and/or disruptions of medical care. Most program directors reported improved patient care as a result.

## Introduction

The COVID-19 pandemic uniquely impacted the medical care of patients with neurodevelopmental disabilities. These patients are at increased risk for impaired motor function, cognition, and social interaction, making them more susceptible to COVID-19 related complications (1). They face significant disruptions to home and school routines, that can cause further declines in health outcomes, and limitations in accessing necessary healthcare and educational services (2, 3), which can further exacerbate their neurodevelopmental impairments. The Center of Disease Control determined that patients with neurological conditions are high-risk and more susceptible to severe complications, when infected with COVID-19 (4). Patients with neurodevelopmental disorders are less able to tolerate mask wearing or observe social distancing requirements, exacerbating the risk of becoming infected with COVID-19 and the stress upon them and their families. The inability of these patients to understand complex situations presents challenges with mood and behavior (5). Inaccessibility to services like physical, occupational, and speech therapies can result in a decline in patient health (6). To bridge this gap, several programs moved to telehealth. This poses an issue for patients struggling to access telehealth services (6), specifically patients from underserved populations (7).

The Kennedy Krieger Institute provides outpatient medical services and care for children and adolescents with disorders involving the brain, spinal cord and musculoskeletal systems. The Kennedy Krieger Institute approaches neurodevelopmental disorders through a multi-professional treatment lens, allowing for expansive integration of changes and improvements in the quality of care. The complex, inter-related factors, unique to patients with neurodevelopmental disorders influence the impact of the COVID-19 pandemic. We hypothesized that clinicians providing medical care during the pandemic would be assisted by this information. We aimed to systematically provide essential COVID-19 pandemic related information to clinicians before their visits with the family, and improve providers ability to deliver high quality care (8).

## Methods

### Participating programs

**Figure 1:**
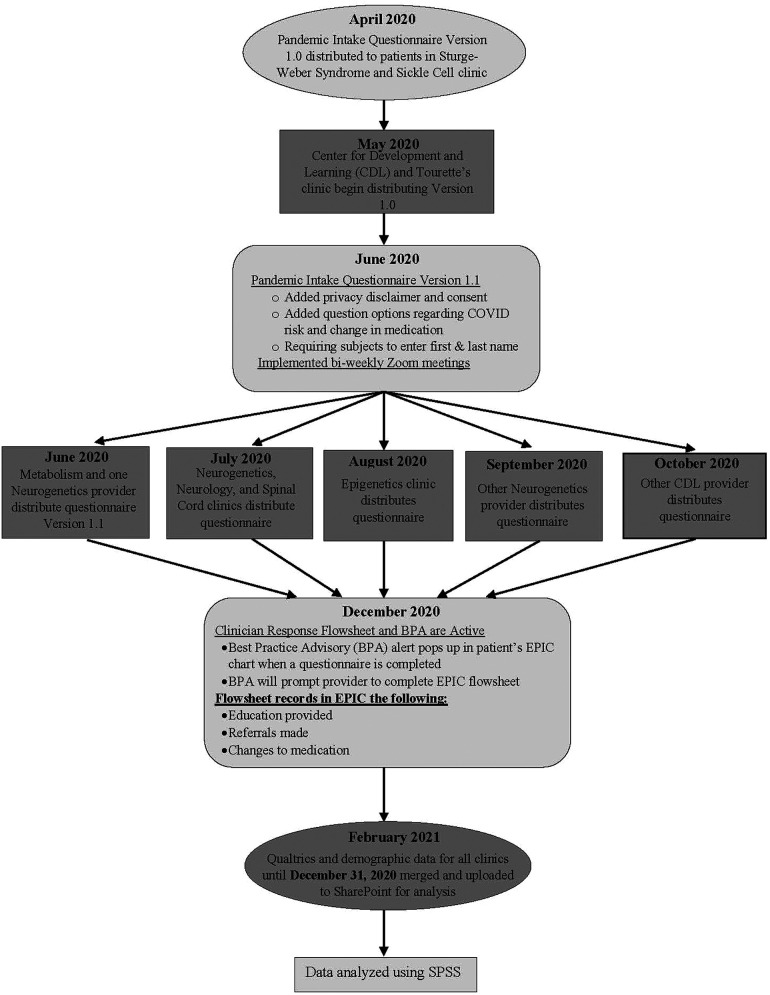
Development and implementation of the pandemic intake questionnaire at the institute during the COVID-19 Pandemic.

This project included 10 clinical programs, led by seven clinician program leaders, including the Center for Development and Learning (CDL), the Center for Autism and Related Disorders (CARD), Tourette's syndrome clinic, Neurogenetics, Neurology, Metabolism clinic, Epigenetics, Sturge-Weber syndrome clinic, Sickle Cell disease clinic, and the Center for Spinal Cord Injury. The Center for Development and Learning (CDL) sees patients diagnosed with cognitive impairments, anxiety, attention deficit hyperactivity disorder, learning disorders, language delays and social difficulties. The Neurology clinic sees patients diagnosed with brain injury, leukodystrophies, Rett syndrome, stroke and epilepsy. Over 4,000 patients (Figure 1) were seen across these programs, during the time of data collection (May 2020 through December 2020). This project received acknowledgement from the Johns Hopkins Institutional Review Board. Pandemic intake questionnaires were accompanied by text (Supplementary Appendix A) which explained to parents that providers would use the data to inform their care at their upcoming visit, and that de-identified information collected could be analyzed. Returning the questionnaire was voluntary and not required for the visit.

### Pandemic intake questionnaire

Clinicians initiated the use of a questionnaire to ask guardians about COVID-19 related concerns prior to clinical visits. This questionnaire was created on the institute's Qualtrics account and distributed *via* web-link to patients prior to their in-person or telehealth clinic visits. The 33 item Pandemic Intake questionnaire collected information regarding the impact that COVID-19 had on their health, medication, and treatment (see Supplementary Appendix A for COVID-19 questionnaire). The Institute's Health Information Management department received the completed questionnaires through automated emails from Qualtrics, and uploaded them to the EPIC health management system. Clinicians viewed the completed questionnaire before or during the visit and addressed COVID-related concerns during the appointment. The Pandemic Intake questionnaire content was implemented beginning in April of 2020 until December 31, 2020 (Figure 1), and included items specific to perceived risk of contracting COVID-19, changes in mood, behavior, sleep, appetite, and access to medical care. Questions used a three point Likert-like scale and guardians indicated if they agreed with the item statement (“yes”), were not sure (“unsure”), or disagreed with the statement (“no”). Questions asked guardians to identify a worsening, improvement, or no change in quality of life due to the COVID-19 pandemic. Free text boxes were included to allow the taker to expand on their concerns.

### Provider feedback survey

To assess the utility of the Pandemic Intake questionnaire, a provider feedback survey was developed and sent to the seven program directors. This assessed whether the directors deemed the Pandemic Intake questionnaire helpful in the care of patients during the pandemic, and how they addressed COVID-related concerns.

### Analysis

Pandemic Intake questionnaire response data were de-identified, linked with Contact Serial Number, and merged with corresponding demographic information from the healthcare record system of EPIC. This de-identified database was stored on a secure Institute shared drive, and analyzed within IBM SPSS 27. Responses on the Pandemic intake questionnaire were dichotomized; for example “Do you consider the patient to be at increased risk for COVID-19 related complications?”, “yes” and “unsure” responses were coded as an affirmative response and “no” was coded as a negative response. Responses were analyzed in relation to sex, age, race/ethnicity and clinic. To increase cell size for statistical analyses, the Epigenetics, Metabolism and Sickle Cell clinics were combined to the category of “Other”, and American Indian/Alaska Native, Native Hawaiian, Other, Other Pacific Islander, and Hispanic races and ethnicities were also grouped together. For patients seen in multiple clinics or multiple times, the first survey answers were analyzed.

Binary logistic regression identified significant predictors of guardian response; the statistical model included sex, age, race, and clinic type as covariates. Race and clinic type were dummy coded, with the largest groups (White children and the Center for Development and Learning, respectively), as the reference groups against which all other groups were compared. Chi-square analysis determined if responses differed between those in more or less medically complicated clinics. A full factorial binary logistic regression (with interaction) determined whether responses differed between group (more vs. less medically complicated) and time of survey completion (early vs. later pandemic), and identified which factors drove statistical significant associations. The clinics noted as medically more complicated include: Neurogenetics, Neurology, Spinal Cord Injury, Sturge-Weber syndrome, Epigenetics, Metabolism and the Sickle Cell clinic. CARD, CDL and the Tourette's clinic were noted as less medically complicated. In the medically more complicated clinics (Table 3), patients were more likely to be treated with several medications simultaneously, have been hospitalized for seizures, surgeries, and other medical issues, and were more likely to be dependent on medical equipment such as wheelchairs, ventilators, and pumps. No formal analysis to attempt to demonstrate these differences was done.

Responses to the provider feedback survey (see Supplementary Appendix B for survey) were organized in an Excel database. They were analyzed within Excel to determine the frequency of answers. The number of providers that found the Pandemic Intake questionnaire helpful in their clinical care was calculated. Changes made to their clinic evaluations in response to the feedback were noted.

## Results

A total of 3,153 questionnaires were returned with a 33.6% response rate (*N* = 1,643; see Table 1). The mean age was 9.5 years (range 0–21.6 years); 1,119 (68.1%) were males and 524 (31.9%) were female. The scale had low internal item inter-correlation, i.e., less than 0.70 (Cronbach's alpha α = 0.491), suggesting that the questionnaire items were sampling from a variety of clinical areas rather than assessing a single, unitary clinical domain (8, 9).

**Table 1:** Clinics and number of survey respondent.

Clinic	Number of Survey Respondents (*n*)
Neurogenetics	76
Center for Autism and Related Disorders (CARD)	82
Neurology	81
Center for Development and Learning (CDL)	1212
International Center for Spinal Cord Injury (ICSCI)	32
Sturge-Weber Syndrome	86
Tourette's Syndrome	36
Other (Epigenetics, Sickle Cell, Metabolism)	38

### Perceived risk of contracting COVID-19

Three hundred and thirty-seven respondents (20.6%) agreed (“yes”) or were “unsure” if their child was at increased risk of contracting COVID-19. 5.7% reported that this was due to asthma/other lung disease and 0.7 reported that was due to heart disease. Out of all respondents, 2.9% reported that their child had asthma/other lung disease and 0.2% reported their child having heart disease at the time of completing the questionnaire. Clinic type was a significant predictor, but sex, age, and race were not (80.5% correctly classified, *χ*^2^ (14, *N *= 1,638)* *=* *158.6, *p *< 0.001). Fifteen percent seen in the Center for Development and Learning reported an increased perceived risk for their children (i.e., “yes” or “unsure”); those from more medically complicated programs, i.e., Spine (59%), Other (58%), Sturge-Weber Syndrome (49%), Neurology (44%), and Neurogenetics (35%) clinics were 3.29–8.77 times more likely to report a perceived increased risk of contracting COVID-19 (Table 2).

**Table 2:** Odds ratios of significant predictors of pandemic intake Questionnaire responses.

COVID-19 Questionnaire Items (Odds Ratios)	Race	Age	Clinic Type
Perceived increased risk of COVID-19	Not Significant	Not Significant	Spine (8.770)
			Other (8.009)
			SWS (6.222)
			Neurology (4.697), Neurogenetics (3.286)
Worsened behavior	Black (0.667)	(0.998)	CARD (2.05)
	Asian (0.486)		Spine (0.12)
Worsened mood	Black (0.484)	(1.003)	Not Significant
	Asian (0.534)		
	Other (0.496)		
Worsened sleep	Not Significant	Not Significant	CARD (2.245)
Worsened appetite	Black (1.922)	(1.006)	SWS (2.382)
Decreased access to therapy	Black (0.60)	(0.993)	Neurogenetics (2.638)
			Other (3.075)
			Spine (12.924)
Decreased access to surgery	Not Significant	Not Significant	Tourette's (3.863)
			Spinal Cord (5.287)
			SWS (7.376)

SWS, sturge weber syndrome; CARD, center for autism and related disorders; other, metabolism, epigenetics, sickle cell clinic.

### Change in mood and behavior

Twenty percent (*n *= 322) reported a worsening in their child's mood during the pandemic; (model correctly classified 80.2% of the sample, *χ*^2^ (14, *N *= 1627) = 43.2, *p *< 0.001). Race and age were significant predictors; sex and clinic type were not. There was a 1.003 cumulative increase of reports in worsened mood with each increase in age by month (*p* = 0.01). Guardians of Black and African American (14%), Other (14%), and Asian (15%) patients were 0.48–0.53 times less likely to report worsening in mood compared to White respondents (24%) (Table 2).

Twenty percent (*n *= 327) of guardians reported worsening in their child's behavior during COVID-19 (model correctly classified 79.9% of the sample, *χ*^2^ (14, *N *= 1,637) = 36.8, *p *= 0.001). Race, age, and clinic type were significant predictors; sex was not. Reports of worsening in behavior during the pandemic was significantly different across race and ethnicity (*p* = 0.002), type of clinic (*p* = 0.014), and across age (*p* = 0.047). Guardians of Black and African American (17%) and Asian (13%) children were 0.667–0.486 times less likely to report worsening in behavior, compared to parents of White children (23%). Twenty percent of guardians of CDL patients reported a worsening of behavior, while guardians of CARD patients (33%) were 2.05 times more likely to report worsening behavior, and respondents from the Spinal Cord clinic (3%) were 0.12 times less likely to report worsening behavior. There was a 0.998 cumulative decrease in reports of worsening behavior with each increase in age by month (Table 2).

### Change in sleep and appetite

Regarding sleep, 11.7% (*n *= 191) indicated the child's sleep worsened during the pandemic (Table 3). The statistical model correctly classified 88.3% of the sample, *χ*^2^ (14, *N *= 1644)* *=* *24.8, *p *= 0.037). Clinic type was a significant predictor; guardians of CARD patients (22%) were 2.2 times more likely to report worsening sleep compared to CDL patients (12%). Sex, race and age were not predictors.

**Table 3:** Comparison of more medically and less medically complicated clinics by pandemic questionnaire item.

	Less Medically Complicated Total	More Medically Complicated Total
Perceived increased risk of COVID-19	9%*	30%*
Worsened behavior	20%	19%
Worsened mood	20%	19%
Worsened sleep	12%	9%
Worsened appetite	5%	4%
Decreased access to therapy	18%*	32%*
Decreased access to surgery	3%*	9%*

*denotes statistical significance in table 3.

Regarding appetite, 4.98% (*n *= 81) reported their child experienced worsened appetite. The statistical model correctly classified 95% of the sample, *χ*^2^ (14, *N *= 1,627) = 28.2, *p *= 0.013). Clinic type, age and race were significant predictors; sex was not. Guardians of patients seen at the SWS center (9%) were 2.4 times more likely to report worsened appetite then CDL patients (5%). Black and African American patients (8%) were 1.9 times more likely to report worsened appetite than White respondents (4%). There was a 1.006 cumulative increase in reports of worsened appetite with increase in age by month (Table 2).

### Difficulty accessing therapy

Twenty percent (*n* = 333) of guardians reported increased difficulty accessing therapies for their children during the pandemic. The statistical model correctly categorized 80.4% of the sample, *χ*^2^ (14, *N *= 1644)* *=* *121.5, *p *< 0.001). Race, age, and clinic type were significant predictors, but not sex. Twenty-three percent (*n *= 200) of guardians of White children indicated increased difficulty accessing therapies; Black and African American respondents (14%) were less likely to report difficulty accessing therapies. Guardians from the Neurogenetics (38%), Other clinics (27%), and the Spinal Cord clinic (72%) were 2.63–12.92 times more likely to report difficulty accessing therapies as compared to 17% (*n* = 206) of those from CDL. There was a 0.993 cumulative decrease in reports of difficulty accessing therapies with each increase in age by month. Finally, 4% (*n* = 66) of guardians indicated difficulty accessing surgical services during the pandemic. The statistical model was significant and correctly categorized 96% of patients, *χ*^2^ (14, *N *= 1,644)* *=* *43.7, *p *< 0.001). Clinic type was a significant predictor; age, sex, and race were not. Respondents from the Tourette's (8%), Spinal Cord (12%), and Sturge Weber syndrome (17%) clinic were 3.86–7.37 time more likely to report difficulty in accessing surgeries compared to respondents from CDL (3%) (Table 2). Those from more medically complicated clinics were more likely to report inability to obtain therapies (32% vs. 18%; *p *< 0.001) and inability to obtain surgeries (9% vs. 3%; *p* < 0.001). A significant main effect for time (early vs. later pandemic) was only observed in the ability to access surgeries over time (*p *= 0.027); more respondents reported difficulty accessing surgeries between May and August (39.4%) than between September and December (13.6%).

### Provider feedback survey

**Figure 2:**
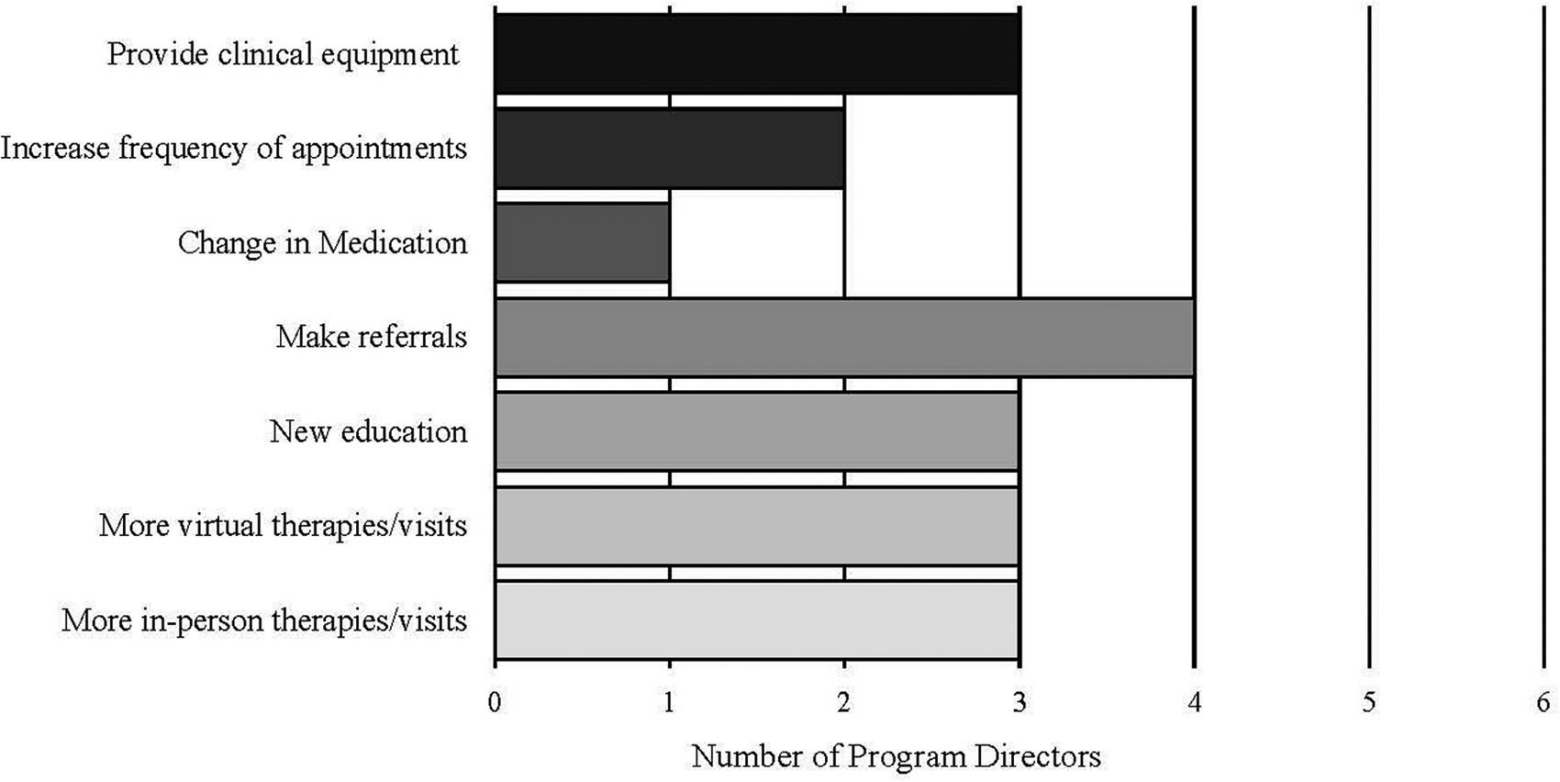
Measures implemented by program directors to address COVID-19 concerns brought up in the pandemic intake questionnaire.

Six of 7 program directors responded to a survey assessing their experience utilizing the Pandemic Intake survey (Table 4). Five out of six found the Pandemic Intake questionnaire helpful in their care of their patients. Most providers reported that changes in sleep (66.7%) and behavior (66.7%) were the most frequent COVID-19 related concerns for their patients. They noted change in overall quality of life (50%) and mood (50%) were frequent concerns. Most directors (66.7%) made more referrals than before COVID-19 pandemic to address these concerns; including referrals to increase training children to tolerate masks, care coordination for resources, and to emergency department/ crisis center for behavioral concerns. Providers also worked to make more in-person (50%) and virtual therapies available (50%), and provided clinical equipment for patients without access (50%) (Figure 2). Providers reported that this process helped identify additional patient concerns specifically with social interactions and school services.

**Table 4:** Results of provider feedback survey.

Clinic Name	Was the Pandemic intake questionnaire helpful?	Briefly, why or why not?	Were any of the following measures/strategies implemented in your program to address COVID-19 patient concerns?							
			Make more in-person therapies/visits available	Make more virtual therapies/visits available	Provide new educational information	Prescribe more/less or different medications	Make more/less /different referrals	Increasing frequency of follow-up appointments	Providing clinical equipment	Other comments
International Clinic of Spinal Cord Injury	Yes	Prepared me to address issues that might not have come up during the virtual visit	Yes	No	No	No	No	No	Yes	
Sickle Cell Disease Clinic	Yes	It has been helpful to know what resources my patients are/are not getting in advance of clinic so I can identify other options for them.	No	Yes	Yes	No	Yes	No	Yes	
Center for Autism & Related Disorders (CARD)	Yes	Helped to assess family stress and also patient's behaviors due to Covid-19	No	Yes	Yes	No	Yes	Yes	No	Increased referrals of families for training children with ASD to tolerate masks. Increase in referrals to Care Coordination for resources
										With Covid I have seen an increase in significant behaviors and also referrals to ED/ Crisis center for patients
Sturge-Weber Syndrome Clinic	Yes	Makes me aware of Covid issues and concerns that need to be discussed.	Yes	Yes	Yes	Yes	Yes	Yes	Yes	
Neurogenetics	No	–	Yes	No	No	No	No	No	No	
Center for Development and Learning (CDL)	Yes	–	No	No	No	No	Yes	No	No	

## Discussion

We successfully implemented a pre-visit Pandemic Intake questionnaire to quickly and systematically inform provider awareness of COVID-19 related patient concerns; the majority of program directors reported that this information helped them provide optimal care. Guardians of patients from more medically complicated clinics were significantly more likely to report perceived increased risk of contracting COVID-19, and the inability to access surgeries and therapies. Patients from CARD had higher reports of worsening behavior. Program directors worked to address these concerns and there was a significant decrease in the number of respondents unable to access surgeries in the later months of the pandemic (September to December). The implementation the Pandemic Intake survey facilitated the exchange of information and ideas, to benefit both patients and providers, and can be adapted to be used as a model for future pandemics at similar institutions.

Perhaps due to their increased risk of co-morbidities (1), parents of patients with neurodevelopmental disorders frequently reported concerns for increased risk of contracting COVID-19. Patients with spinal cord injuries (10), sickle cell disease (11), developmental (12), and neurological (8) conditions are reported to be at increased risk of COVID-19 infection. Program directors made more appointments available and provided COVID-19 education to their patients. Telemedicine is a safe and satisfactory alternative to in-person appointments and encourages patients to discuss their concerns relating to COVID-19 risk (13). There was no difference in perceived increased risk of contracting COVID-19 across race, age and sex; this data contradicts previously reported information where Black and Asian individuals were at higher risk (14). However, these other data were not from children with neurodevelopmental disorders. For patients at the Kennedy Krieger Institute, clinical care requires access to physical, occupational and speech therapy. Due to the pandemic, many of these services transitioned to telerehabilitation (15). The data collected suggests that patients from medically complicated clinics were more reliant on therapies and were more affected by the inability to access it. Providers responded by making more in-person and virtual appointments available to increase access. Reports for difficulty in accessing surgeries decreased over time.

The pandemic's stay-at-home order significantly impacted mental health, routines and quality of life. Reports show higher rates of anxiety, depression, psychological distress and stress (16). Limitations in social interaction impacted the quality of life, and worsening behavior from patients at the Center for Autism and Related Disorders (CARD). Aggression, hypersensitivity and behavioral problems are issues in patients with autism spectrum disorder. The CARD program director reported making more referrals to the crisis center and care coordination to increase resources and address these behavioral changes (17). This study reports worsened sleep in patients at CARD; previous research found patients with ADHD and ASD to report high rates of delays in bedtime, decreased sleep duration and increased sleep disorders (18). Virtual learning, increase stress, and altered routines impacted the sleep and appetite patterns of children during the pandemic (17). This study suggests that patients from the Sturge-Weber clinic and patients that were Black had higher reports of worsening appetite. Providers addressed this concern by making referrals to other clinics.

This study has important limitations. While the overall process of disseminating questionnaires was the same across programs, there were differences in the details of execution, like, in how many days before the clinic appointment the questionnaires were sent, which may have impacted participation. Families with infrequent access to email and non-English speaking families may have been less likely to participate. This was a cross-sectional study; further work is needed to understand implementation over time during the pandemic.

## Conclusion

This approach, and Pandemic Intake Questionnaire, may prove useful as the COVID-19 pandemic situation evolves, and for future pandemics, both at the Kennedy Krieger Institute and at other similar medical facilities.

## Data Availability

The raw data supporting the conclusions of this article will be made available by the authors, without undue reservation.
