# Supplementary material for: Pandemic intake questionnaire to improve quality, effectiveness, and efficiency of outpatient neurologic and developmental care at the Kennedy Krieger institute during the COVID-19 pandemic
Source: Front Rehabil Sci. 2022 Oct 6;3:934558. doi: 10.3389/fresc.2022.934558 (PMC9583877; doi:10.3389/fresc.2022.934558)
Supplement: Supplementary file 1 [file DataSheet1.pdf]

Survey

Kennedy Krieger Institute

COVID-19 Patient Questionnaire

We look forward to seeing you at your upcoming appointment at the Kennedy Krieger Institute. We are committed to providing you with the best medical experience possible. Your answers to these questions in this unique situation will help your provider better prepare for your visit and meet your medical needs. Please fill out these questions on behalf of the patient. If you are uncertain about how to answer a question, please let your provider know. Please allot

5-10 minutes to complete these questions. Thank you for your time.

At Kennedy Krieger Institute, we are committed to protecting your privacy, providing high quality care, and constantly improving. The responses you provide on this survey will help us provide your child with the best care possible. Your responses will also help us learn more about what we can do to improve the care that other children receive at Kennedy Krieger Institute. By completing this survey, you are giving permission for us to look at your responses to improve our services, we will keep your information confidential by removing names, birthdates, and other identifying information. Thank you for helping us improve the care that current and future patients receive at the Kennedy Krieger Institute.

Please be advised that the information you provide to us may not be viewed by someone at Kennedy Krieger Institute until the day of your child's appointment. **If you or your child are experiencing a medical or mental health emergency, please contact 911 or go to the nearest emergency room for immediate assistance.**

What is the patient's first and last name?

What is the patient's date of birth?

What is your relationship to Patient's Name ?

What is your relationship to Patient's Name ?

Has Patient's Name , or a family member, been suspected to have COVID-19?

- Yes
- No
- Suspected

Please tell us what happened

Do you consider Patient's Name to be at increased risk for COVID-19 related complications?

- ○ Yes
- ○ No
- ○ Unsure

Why do you believe Patient's Name is at increased risk of COVID-19? Please select all that apply.

- ☐ Asthma/Other Lung Disease
- ☐ Heart Disease
- ☐ Diabetes
- ☐ Immune Suppression
- ☐ Other

Does Patient's Name , , have any of the following?

- ☐ Asthma/Other Lung Disease

- [ ] Heart Disease
- [ ] Diabetes
- [ ] Immune Suppression
- [ ] Other

Please explain why Patient's Name is at increased risk of COVID-19:

Please describe the other condition:

Have you changed Patient's Name medications because of the COVID-19 crisis?

- ( ) Yes

○ No

How was the medication changed?

- ☐ Added a medication
- ☐ Stopped a medication
- ☐ Changed when or how a medication is given
- ☐ Taken a medication more regularly
- ☐ Other medication change not listed above

Please explain in what way the medication was changed:

Please explain why the medication was changed:

Has Patient's Name , , had a change in neurologic status (such as headaches, seizures control, or motor function) because of the COVID-19 crisis?

- ◯ Yes
- ◯ No

What is the change in neurologic status?

- ◯ Improved
- ◯ Worsened

Please explain the change in neurologic status occurring because of COVID-19

How have the following been impacted by the COVID-19 crisis for Patient's Name ?

	No Change	Improved	Worsened

	No Change	Improved	Worsened
Sleep	○	○	○
Appetite	○	○	○
Mood	○	○	○
Behavior	○	○	○

Please explain how COVID-19 has affected Patient's Name 's sleep, appetite, mood, or behavior:

Please tell us whether the COVID-19 crisis has improved, worsened or did not change Patient's Name 's overall quality of life.

- ○ Severe Worsening
- ○ Moderate Worsening
- ○ Mild Worsening
- ○ No Change
- ○ Mild Improvement
- ○ Moderate Improvement

○ Great Improvement

Please explain what ways overall quality of life has changed:

Where have you obtained most of your information related to COVID-19 and how to care for Patient's Name ? Please check all that apply.

- ☐ My Doctors and Other Providers
- ☐ The CDC (Centers for Disease Control and Prevention)
- ☐ Social Media
- ☐ Parent Advocacy/Support Groups
- ☐ Online News/Media Outlets
- ☐ Other

From what other sources have you obtained most of your information related to COVID-19, and how to care for Patient's Name ?

What medical needs have been unmet during the COVID-19 crisis? Please check all that apply.

- ☐ Difficulty Obtaining Medications
- ☐ Inability to Obtain Therapies
- ☐ Inability to Obtain Needed Surgeries or Procedures
- ☐ Inability to Access Primary Care Providers
- ☐ Inability to Access Specialists
- ☐ Other
- ☐ All of My Needs Have Been Met

Please explain how medical needs have been unmet:

Please share any additional comments or concerns about how the COVID-19 crisis has impacted [illegible] Patient's Name}'s medical situation.

Powered by Qualtrics
