# Supplementary material for: Pandemic intake questionnaire to improve quality, effectiveness, and efficiency of outpatient neurologic and developmental care at the Kennedy Krieger institute during the COVID-19 pandemic
Source: Front Rehabil Sci. 2022 Oct 6;3:934558. doi: 10.3389/fresc.2022.934558 (PMC9583877; doi:10.3389/fresc.2022.934558)
Supplement: Supplementary file 2 [file DataSheet2.pdf]

Default Question Block

Kennedy Krieger Institute

COVID-19 Provider Feedback Survey

Block 4

Name

Clinic

Block 1

Has the COVID-19 intake questionnaire been helpful to you in the care of your patients during the COVID-19 pandemic? Please indicate only ONE of the responses below:

- Yes
- No
- Unsure

Briefly, why or why not?

Block 2

From your review of the patient surveys, what has been your impression of the greatest COVID-19 related concern/s for your patients?
Please indicate all that apply:

- ☐ Change in Medication
- ☐ Change in Neurologic Status
- ☐ Change in Sleep
- ☐ Change in Appetite
- ☐ Change in Mood
- ☐ Change in Behavior
- ☐ Change in Overall Quality of Life
- ☐ Inability to Access/Obtain Medication
- ☐ Inability to Access/Obtain Therapies
- ☐ Inability to Access/Obtain Surgeries/Procedures
- ☐ Inability to Access/Obtain PCPs
- ☐ Inability to Access/Obtain Specialists
- ☐ Other

If other, please describe:

Block 3

What new measures/strategies have you implemented in your program to address these concerns in response to COVID-19?
Please indicate all that apply.

- ☐ Work to make more in-person therapies/visits available to patients
- ☐ Work to make more virtual therapies/visits available to patients
- ☐ Provide new educational information on COVID-19 not typically part of your clinical visit
- ☐ Prescribe medications that you did not typically prescribe pre-COVID, or change the frequency of medications prescribed
- ☐ Make referrals that you did not typically make pre-COVID, or change the frequency of referrals made
- ☐ Increasing frequency of follow-up appointments
- ☐ Providing clinical equipment for patients without access (iPads, scales, blood pressure monitor, etc.)
- ☐ Other

If other, please describe:
